# Supplementary material for: Magnetic Resonance Imaging Measurement of Entorhinal Cortex in the Diagnosis and Differential Diagnosis of Mild Cognitive Impairment and Alzheimer’s Disease
Source: Brain Sci. 2021 Aug 26;11(9):1129. doi: 10.3390/brainsci11091129 (PMC8471837; doi:10.3390/brainsci11091129)
Supplement: Supplementary file 1 [file brainsci-11-01129-s001.zip › brainsci-1286478-supplementary.pdf]

Supplementary Materials: Magnetic Resonance Imaging Measurement of Entorhinal Cortex in the Diagnosis and Differential Diagnosis of Mild Cognitive Impairment and Alzheimer's Disease

Qianqian Li [1,2,†], Junkai Wang [3,†], Jianghong Liu [4], Yumeng Wang [5] and Kuncheng Li [1,2,*]

Table S1. The AUCs of thickness, surface area and volume of ERC.

	Thickness		Surface area		Volume	
	**right**	**left**	**right**	**left**	**right**	**left**
HC vs aMCI-s	0.558	0.711	0.580	0.536	0.684	0.639
HC vs aMCI-m	0.736	0.729	0.553	0.510	0.774	0.778
HC vs AD	0.908	0.876	0.579	0.505	0.863	0.860
aMCI-s vs aMCI-m	0.647	0.505	0.513	0.520	0.589	0.527
aMCI-s vs AD	0.824	0.711	0.541	0.510	0.724	0.711
aMCI-m vs AD	0.694	0.707	0.519	0.514	0.654	0.697

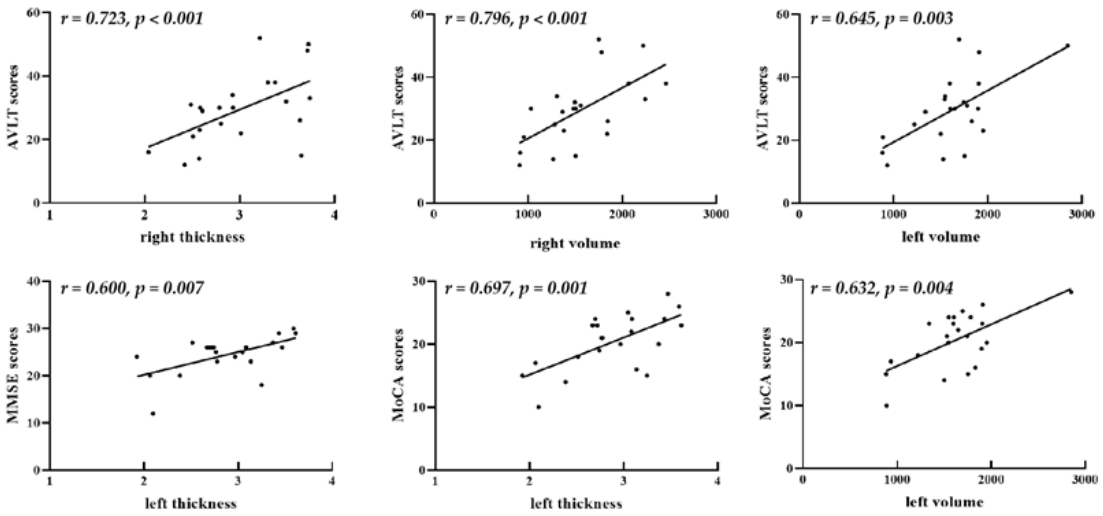

Figure S1. The relationship between ERC morphometric measurements and memory performance in aMCI-m group. Note: thickness and volume of ERC were measured in millimeters (mm) and cubic millimeters (mm^3) respectively.

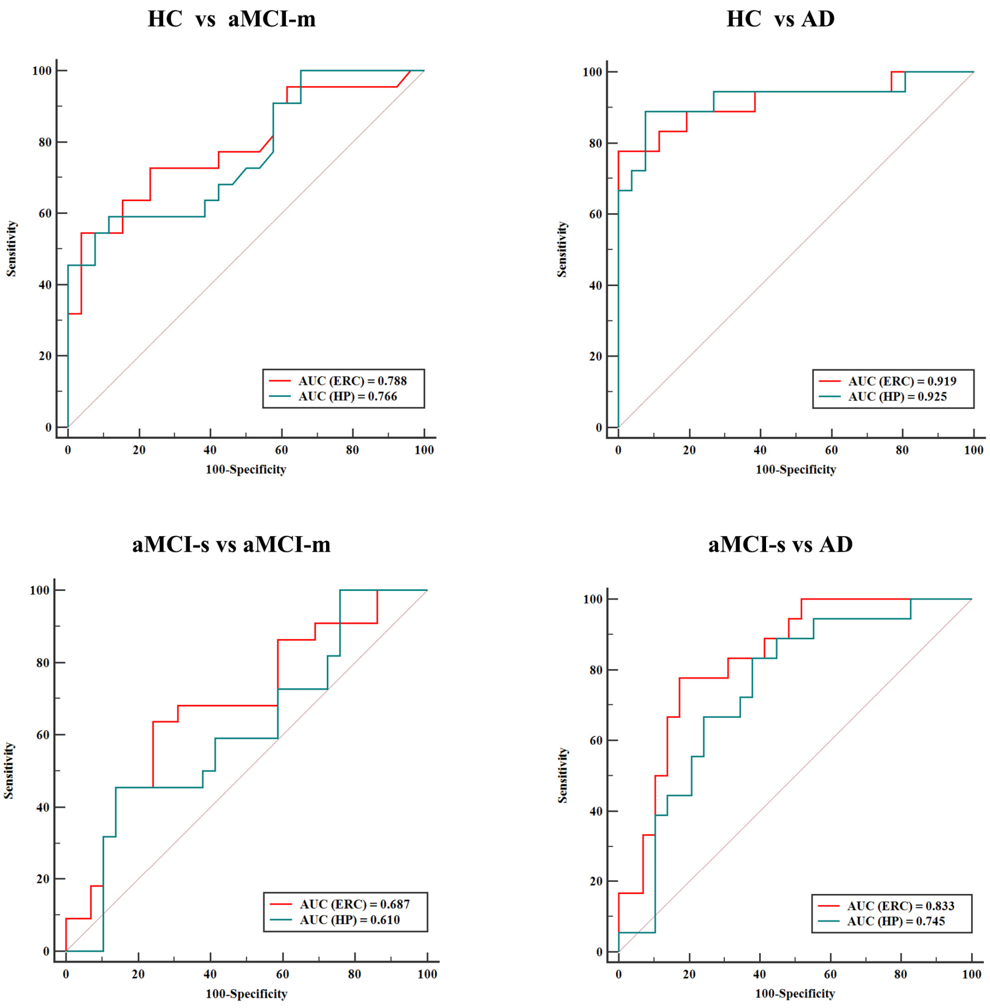

Figure S2. The ERC vs HP in discriminating HC from aMCI-m, HC from AD, aMCI-s from aMCI-m and aMCI-s from AD. Red: entorhinal cortex; Green: hippocampus.
